# Genome Wide Meta-Analysis identifies common genetic signatures shared by heart function and Alzheimer’s disease

**DOI:** 10.1038/s41598-019-52724-2

**Published:** 2019-11-13

**Authors:** M. E. Sáez, A. González-Pérez, B. Hernández-Olasagarre, A. Beà, S. Moreno-Grau, I. de Rojas, G. Monté-Rubio, A. Orellana, S. Valero, J. X. Comella, D. Sanchís, A. Ruiz

**Affiliations:** 1 Andalusian Bioinformatics Research Centre (CAEBi), Seville, Spain; 2 Research Center and Memory Clinic, Fundació ACE. Institut Català de Neurociències Aplicades-Universitat Internacional de Catalunya (UIC), Barcelona, Spain; 3 Universitat de Lleida – IRBLleida, Lleida, Spain; 4 Centro de Investigación Biomédica en Red sobre Enfermedades Neurodegenerativas (CIBERNED), ISCIII, 28031 Madrid, Spain; 5 Institut de Recerca Hospital Universitari de la Vall d’Hebron (VHIR), Barcelona, Spain

**Keywords:** Disease genetics, Molecular medicine

## Abstract

Echocardiography has become an indispensable tool for the study of heart performance, improving the monitoring of individuals with cardiac diseases. Diverse genetic factors associated with echocardiographic measures have been previously reported. The impact of several apoptotic genes in heart development identified in experimental models prompted us to assess their potential association with human cardiac function. This study aimed at investigating the possible association of variants of apoptotic genes with echocardiographic traits and to identify new genetic markers associated with cardiac function. Genome wide data from different studies were obtained from public repositories. After quality control and imputation, a meta-analysis of individual association study results was performed. Our results confirmed the role of caspases and other apoptosis related genes with cardiac phenotypes. Moreover, enrichment analysis showed an over-representation of genes, including some apoptotic regulators, associated with Alzheimer’s disease. We further explored this unexpected observation which was confirmed by genetic correlation analyses. Our findings show the association of apoptotic gene variants with echocardiographic indicators of heart function and reveal a novel potential genetic link between echocardiographic measures in healthy populations and cognitive decline later on in life. These findings may have important implications for preventative strategies combating Alzheimer’s disease.

## Introduction

Echocardiographic assessment of cardiac structure offers prognostic information about cardiac conditions such as heart failure (HF)^1,2^. Pathological processes including cardiomyocyte cell death, inflammatory cell response and changes in interstitial tissue of the heart are factors leading to adverse remodelling and HF^3^.

A number of apoptotic genes have been investigated as potential targets to prevent cardiomyocyte death, but it is now increasingly evident that caspase-dependent cell death plays a minor if any role in adult myocyte loss^4^, which involves Cyclophilin D^5^ and calpains^6^. By contrast, caspase proteins are now recognized as important factors for initial differentiation of stem cells to cardiomyocytes^7^, and its deficiency *in vivo* was shown to induce abnormal heart development^8,9^. In rodent cardiomyocytes, caspase-3 is involved in WNT signalling and myocyte growth^10,11^, contributing also to muscle-specific gene splicing by cleaving PTB^12^. In addition, apoptotic DNA nucleases were shown to play a role in the developmental process of *C.elegans* including the *C.elegans* Caspase-associated DNase (CAD), ENDOG and TATD orthologues^13^. Furthermore, ENDOG also contributes to the signalling pathways determining myocyte size through the control of reactive oxygen radicals (ROS)^14,15^. These facts lead us to hypothesize that caspases and the nucleases ENDOG and TATD play relevant functions in cardiomyocyte proliferation and maturation during development.

Recent genome wide association studies have been performed for evaluating comprehensive sets of echocardiographic traits in well characterized individuals included in large cohort studies^16,17^. Increased left ventricular mass (LVM) is a well-established predictor of adverse cardiovascular events and premature death^18,19^. End-Diastolic LV Internal Dimension (LVID) and LV Wall Thickness (LVWT) are other measures of left ventricular hypertrophy (LVH), usually a pathological compensatory mechanism of the LV overload which evolves towards progressive LV dysfunction and HF. The principal role of the left atrium (LA) is to modulate LV filling and cardiovascular performance, being LA enlargement and dysfunction also predictors of cardiovascular events, especially in patients with atrial fibrillation (AF)^20^. The risk of aortic aneurysm is strictly related to the diameter of the ascending aorta, and recent studies have shown high prevalence of aortic root (AROT) enlargement in the hypertensive population^21^. Using data from publicly available repositories, we aimed to explore the association between a selected group of candidate apoptosis-related genes and these echocardiographic phenotypes by means of meta-GWAS. Furthermore, we aimed to assess previously reported signals in our study datasets and performed an agnostic analysis to investigate relevant pathways revealed for each trait. Following the leading results of this analysis, we further explored the unsuspected genetic relationship between Alzheimer’s disease (AD) and these echocardiographic traits, by estimating their genetic correlation, and identifying common genetic determinants of these conditions.

## Materials and Methods

### Experimental design

This study is aimed at identified genetic variants associated with echocardiographic traits, using both a candidate gene approach and an agnostic approach in retrospective datasets available in public repositories.

### Study cohorts

The four cardiovascular datasets analysed in this study were downloaded from dbGAP (https://www.ncbi.nlm.nih.gov/gap) after requesting the appropriate permissions. In the case of a multi-ethnic study, only Caucasian samples after principal component analysis (PCA) were retained for analysis. A summary of the clinical characteristics of these populations is shown in Table 1. A full description of each of them is provided in the Supplementary Info.

**Table 1:** Study sample characteristics.

Dataset	TRAIT (units)	All		Males		Females	
		MEAN (SD)	MIN/MAX	MEAN (SD)	MIN/MAX	MEAN (SD)	MIN/MAX
CARDIA (N = 1362, 640 males, 722 females)	AGE (yrs)	25.51 (3.33)	17/32	25.59 (3.27)	17/32	25.44 (3.38)	18/31
	BMI (kg/m^2^)	23.71 (4.04)	16.28/45.09	24.43 (3.60)	16.81/43.17	23.07 (4.31)	16.28/45.09
	AROT (cm)	2.81 (0.39)	1.67/4.27	3.04 (0.36)	1.67/4.27	2.61 (0.30)	1.88/3.68
	LAS (cm)	3.52 (0.46)	2.11/5.24	3.69 (0.44)	2.39/5.19	3.37 (0.41)	2.11/5.24
	LVID (cm)	4.99 (0.48)	3.66/7.05	5.24 (0.44)	3.99/7.05	4.76 (0.40)	3.66/6.12
	LVM (g)	157.53 (47.08)	49.85/364.91	145.44 (42.00)	63.38/364.91	147.44 (44.55)	58.06/328.80
	LVWT (cm)	1.68 (0.25)	1.01/3.30	1.80 (0.24)	1.17/2.96	1.57 (0.22)	1.01/3.30
CHS (N = 2988, 1178 males, 1810 females)	AGE (yrs)	72.42 (5.47)	64/100	73.09 (5.72)	65/100	71.98 (5.26)	64/97
	BMI (kg/m^2^)	26.36 (4.47)	14.65/49.41	26.38 (3.64)	15.80/46.23	26.35 (4.94)	14.65/49.41
	AROT (cm)	3.15 (0.46)	1.54/4.38	3.45 (0.43)	1.54/4.83	2.96 (0.36)	1.84/4.54
	LAS (cm)	3.84 (0.65)	1.77/8.78	4.00 (0.66)	1.81/8.78	3.73 (0.61)	1.77/6.50
	LVID (cm)	4.90 (0.65)	3.01/8.27	5.20 (0.68)	3.28/8.27	4.72 (0.56)	3.01/6.98
	LVM (g)	146.81 (46.45)	58.12/435.70	172.14 (50.96)	70.90/435.70	131.85 (36.00)	58.12/392.88
	LVWT (cm)	1.72 (0.29)	0.96/3.92	1.81 (0.32)	1.12/3.92	1.67 (0.26)	0.96/3.12
FHS (N = 2668, 1224 males, 1444 females)	AGE (yrs)	33.61 (9.29)	5/60	33.50 (9.38)	11/60	33.70 (9.22)	5/59
	BMI (kg/m^2^)	24.91 (4.13)	13.52/50.98	26.27 (3.60)	13.52/43.63	23.76 (4.21)	15.02/50.98
	AROT (cm)	3.14 (0.40)	2.00/4.90	3.38 (0.37)	2.00/4.90	2.94 (0.30)	2.00/4.20
	LAS (cm)	3.72 (0.51)	2.20/6.00	3.94 (0.47)	2.50/6.00	3.52 (0.46)	2.20/5.70
	LVID (cm)	4.84 (0.47)	2.70/7.20	5.09 (0.43)	3.10/7.20	4.63 (0.39)	2.70/6.00
	LVM (g)	168.15 (52.67)	64.33/593.40	194.29 (53.57)	85.07/593.40	146.23 (40.52)	64.36/392.08
	LVWT (cm)	1.88 (0.25)	0.84/3.90	1.96 (0.24)	0.84/3.90	1.80 (0.22)	0.85/2.90
MESA (N = 2379, 1151 males, 1210 females)	AGE (yrs)	61.64 (10.18)	39/96	61.4 (10.36)	39/86	61.90 (10.01)	44/96
	BMI (kg/m^2^)	28.65 (5.67)	15.36/65.28	28.41 (5.44)	15.36/54.50	28.86 (5.85)	15.68/65.28
	AROT (cm)	3.20 (0.38)	0.84/1.48	3.36 (0.38)	1.72/4.98	3.07 (0.33)	1.64/4.86
	LAS (cm)	NA	NA	NA	NA	NA	NA
	LVID (cm)	4.48 (0.57)	2.33/7.39	4.58 (0.60)	2.33/7.36	4.39 (0.53)	2.54/7.39
	LVM (g)	120.16 (29.27)	47.85/315.08	137.42 (27.35)	61.58/315.08	104.16 (20.63)	47.85/227.49
	LVWT (cm)	1.92 (0.40)	0.85/3.93	2.09 (0.39)	0.90/3.93	1.76 (0.33)	0.85/3.56

BMI: Body Mass Index; AROT: aortic root; LAS: Left Atrial Size; LVID: Left Ventricular Internal Dimension; LVM: Left Ventricular Mass; LVWT: Left Ventricular Wall Thickness.

A total of seven AD datasets were used to further explore the observed enrichment of top genes derived from the analysis of echocardiographic traits on genes involved in AD pathways. As for previously described datasets, only Caucasian samples after principal component analysis (PCA) were retained for analysis. Demographic characteristics of these datasets are summarized in the Supplementary Table 1.

### Phenotypes

Data from the most recent available echocardiographic examinations of each cohort were included in this study. The following five phenotypes were analysed: LVM (g), AROT (cm), LVID (cm), LA size LAS, cm), and LVWT (cm). The latter was defined as the sum of the End-Diastolic Thicknesses of the Posterior Wall (TPW) and End-Diastolic Thicknesses of the Interventricular Septum (TIS). LVM was calculated using the formula 0.8 [1.04{(LVID + TIS + TPW)3 −(LVID)3}] + 0.6^22^.

### Genotyping and imputation

The cardiovascular datasets included in this study were genotyped using different platforms: CARDIA and MESA were genotyped using the Affymetrix Genome-Wide Human 6.0 array, whereas the FHS was genotyped using the Affymetrix Human 500k array and the CHS cohort with the Illumina HumanCNV370-Duo v1.0.

AD datasets were genotyped using the Illumina arrays Human 610‐Quad BeadChip (ADNI1, AddNeuroMed batch 1), the HumanOmniExpress BeadChip (ADNI2/GO, AddNeuroMed batch 2, ADGC dataset 3), the Human660W-Quad (ADGC datasets 1 and 2) and the HumanHap300-Duo BeadChip (The Mayo study) or the Affymetrix 250k NspI (the Neocodex-Murcia study), 500k (the TGEN and GenADA studies) or 6.0 (ROSMAP study) arrays.

Prior to imputation, we first performed an extensive quality control excluding individuals with more than 3% missing genotypes, with excess autosomal heterozygosity (>0.35), those showing a discrepancy between genotypic and reported sex, as well as individuals of non-European ancestry based on PCA using SMARTPCA^23^. At the genotype level, we removed single nucleotide polymorphisms (SNPs) with missing genotype rate > 5%, not in Hardy-Weinberg equilibrium (p < 10^−6^) and SNPs with minor allele frequency (MAF) < 1%. Duplicated and related individuals were identified and removed by means of Identity By State (IBS) estimates within and across studies.

Genotype imputation is aimed at estimating unobserved genotypes using as reference known haplotypes from a well characterized population. Imputation was performed using the minimac3 algorithm and the SHAPEIT tool for haplotype phasing at the University of Michigan server using the HRC reference panel^24^. After imputation, SNPs with an R2 quality estimate lower than 0.3 were excluded from further analyses in accordance with software instructions.

### Statistical analysis

All analyses were performed in Caucasian populations only. Individuals with prevalent myocardial infarction (MI) or congestive heart failure (CHF) were excluded from the study. Linear regression models available from PLINK software^25^ were fitted to investigate the association between genotypes and quantitative phenotypes, with age, sex, body mass index and the four principal components as covariates. For each phenotype, we obtained summary estimates of association across studies by using a fixed-effects model meta-analysis procedure implemented also in PLINK. For the genome wide SNP analysis, the conventional GWAS significance threshold was used (p = 5 × 10^−8^)^26^, whereas signals with p value < 10^−5^ were considered as suggestive of association and reported in the Supplementary Tables 2–6.

Gene-wise statistics were computed using MAGMA software, which takes into account physical distance and linkage disequilibrium (LD) between markers to estimate a summary gene p-value using a known approximation of the sampling distribution^27^. All SNPs with MAF above 1% were used in these analyses. At each trait, genes were ranked according to the global p mean value.

Top results from MAGMA genome wide analyses were tested for over-representation of genes involved in known pathways, functions and diseases. For these enrichment analyses, we applied a moderated threshold of p < 10^−3^, which has been showed to have a good power for detecting relevant pathways and functions while maintaining type 1 error controlled^27^. For these analyses, we used the R packages Webgestalt (GO terms, KEGG, Wikipathways and Reactome) and enrichR^28,29^ (Aging perturbations from GEO, Biocarta, Panther Disease perturbations from GEO Disease signatures from GEO, dbGaP and OMIM) with default parameters.

In order to explore genetic correlation between different traits we used a bivariate GREML analysis with GTCA software^30^. This method allows to identify pleiotropic gene effects associated with different diseases or disorders, providing a single measure of the proportion of shared genetic determinants. Furthermore, we obtained summary estimates of association across phenotypes performing unweighted meta-analysis of Fisher p-values.

## Results

**Figure 1:**
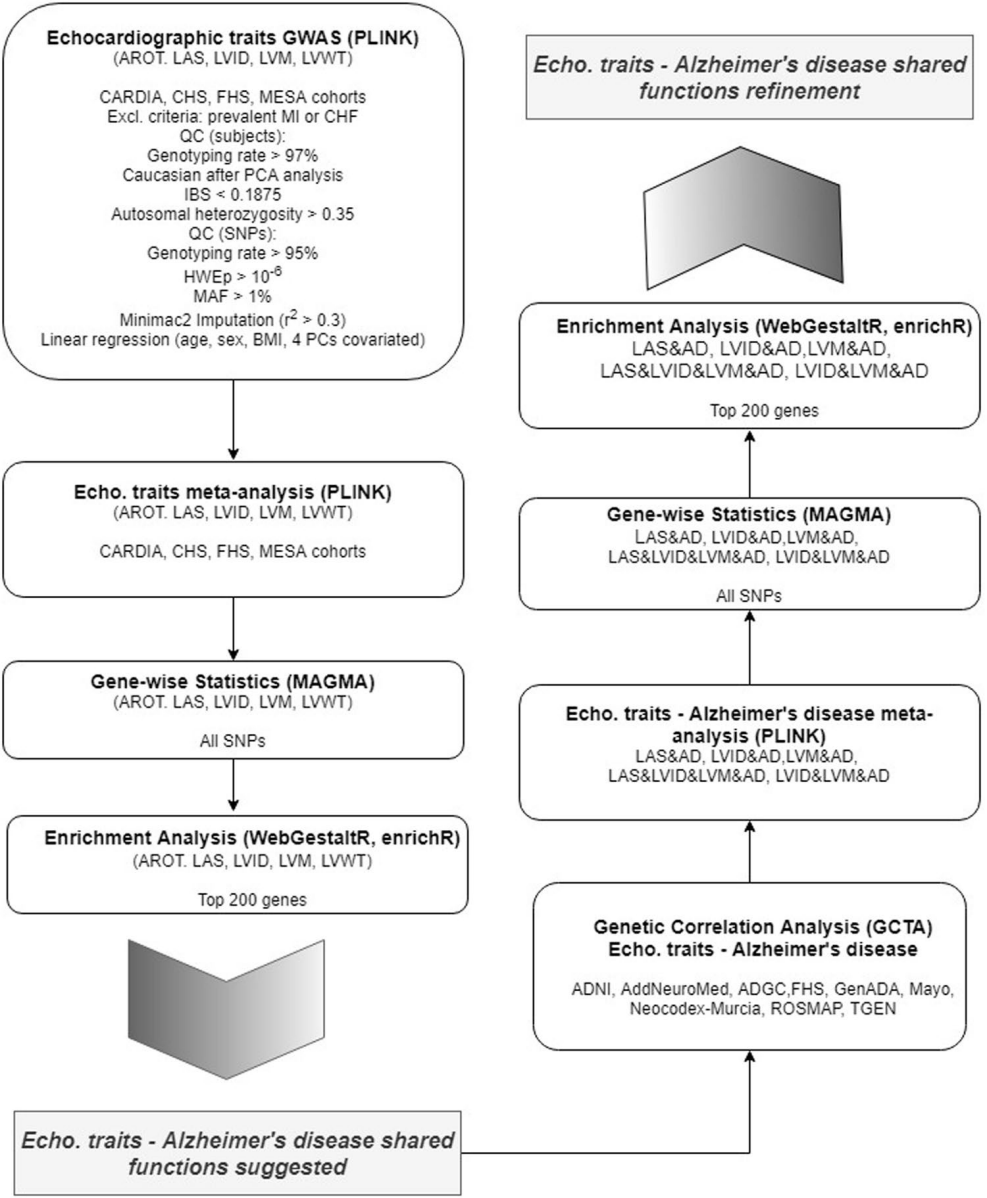
Data analysis workflow.

**Figure 2:**
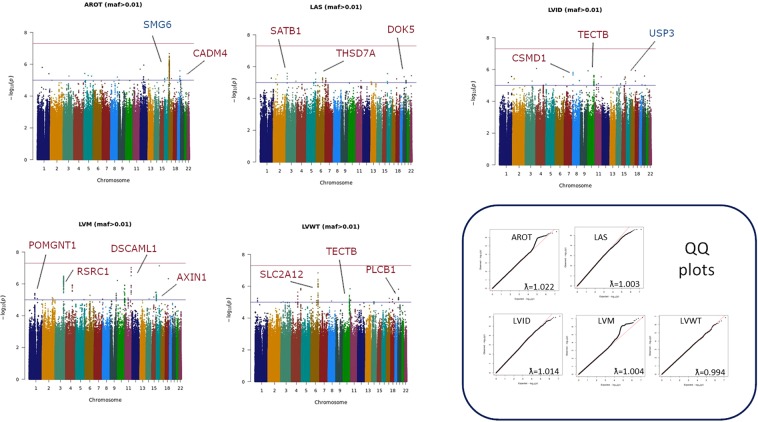
Manhattan plots of the meta-analyses for the different echocardiographic traits. The threshold for genome-wide significance (P < 5 × 10^−8^) is indicated by the red line, while the blue line represents the suggestive threshold (P < 1 × 10^−5^). Loci previously associated with echocardiographic traits are shown in blue, and newly associated loci are shown in red.

Overall, our study included data from 11,559 individuals with echocardiographic phenotypes from four different datasets (Table 1). Figure 1 illustrates the data analysis roadmap as explained in Materials and Methods. After imputation and quality control, we obtained about 7 million SNPs with MAF > 0.01 that were tested for association with echocardiographic traits at each study. We then performed a meta-GWAS to obtain summary estimates of association for each SNP. Genomic inflation factor (λ) ranged from 0.994 to 1.022 in these analyses, indicating absence of population stratification due to hidden population structure (Fig. 2). MAGMA software was used for summarizing the meta-GWAS SNP results in order to obtain a gene-wise statistic of the association between 18,480 genes and the five phenotypes.

### Association of apoptosis-related genes with cardiac phenotypes

Because there is experimental evidence supporting the role of some apoptosis-related genes with cardiac development and disease, we first analysed the potential association of polymorphisms in a series of apoptosis-related genes with the cardiac phenotypes. Table 2 shows the association results of the 20 apoptosis-related candidate genes. Study-wide statistically significant results were observed for the association of a genetic locus on 2q33.1 involving two initiator caspases (*CASP8* and *CASP10*) and the apoptosis regulator protein *CFLAR* (CASP8 And FADD Like Apoptosis Regulator, c-FLIP) with LVM. The same three genes were also linked to LVID with p < 0.05, along with the Fas receptor-associated adaptor *FADD* (Fas-associated protein with death domain) and BCL2 (B-cell lymphoma-2) genes. *BCL2, FADD* and *TATDN1* (TatD DNase domain containing-1) showed a trend for association with AROT. We did not find evidence of association of any calpain family member with any of the analysed traits (data not shown).

**Table 2:** Candidate genes for echocardiographic traits.

GENE	CHR	START	STOP	REGION	NSNPS	NPARAM	AROT p-value	LAS p-value	LVID p-value	LVM p-value	LVWT p-value
*BCL2*	18	60590579	61187011	18q21.33	1760	211	0.0020^*^	0.7439	0.0366^*^	0.1299	0.2162
*CASP1*	11	104696235	105105884	11q22.3	1059	71	0.6532	0.4927	0.7579	0.1082	0.2240
*CASP2*	7	142785308	143204789	7q34	831	101	0.7667	0.7005	0.8837	0.9213	0.6935
*CASP3*	4	185348850	185770629	4q35.1	1373	100	0.8597	0.0895	0.7466	0.3404	0.7148
*CASP4*	11	104613594	105039325	11q22.3	1159	80	0.5809	0.6457	0.8445	0.1983	0.2090
*CASP5*	11	104664967	105093895	11q22.3	1121	74	0.6527	0.5702	0.7841	0.1146	0.1843
*CASP6*	4	110409785	110829814	4q25	981	89	0.7720	0.2404	0.6319	0.6570	0.2016
*CASP7*	10	115238921	115690668	10q25.3	1276	104	0.7140	0.0763	0.6045	0.8870	0.5393
*CASP8*	2	201898166	202352434	2q33.1	970	81	0.3781	0.2791	0.0272^*^	3.20 × 10^−5**^	0.1006
*CASP9*	1	15617896	16051407	1p36.21	1184	87	0.6575	0.2219	0.6237	0.0920	0.7480
*CASP10*	2	201847621	202294129	2q33.1	961	82	0.4802	0.2476	0.0242^*^	5.76 × 10^−5**^	0.0787
*CASP12*	11	104556445	104969397	11q22.3	998	78	0.6042	0.6827	0.7403	0.1616	0.3387
*CASP14*	19	14960291	15369104	19p13.12	1751	162	0.9466	0.4207	0.3398	0.2161	0.2630
*CFLAR*	2	201780877	202237411	2q33.1	926	76	0.6599	0.1414	0.0243^*^	1.07 × 10^−4**^	0.0522
*ENDOG*	9	131380779	131784955	9q34.11	496	43	0.7003	0.6451	0.6393	0.1198	0.6389
*FADD*	11	69849269	70253508	11q13.3	1293	122	0.0029^*^	0.9020	0.0398^*^	0.1055	0.6117
*FAS*	10	90550288	90975542	10q23.31	1352	137	0.4230	0.3532	0.5120	0.6042	0.8215
*TATDN1*	8	125300735	125751329	8q24.13	1421	137	0.0084^*^	0.7526	0.0766	0.5603	0.7433
*TATDN2*	3	10090177	10522906	3p25.3	1340	114	0.7445	0.8405	0.7814	0.1642	0.6076
*TATDN3*	1	212765170	213190167	1q32.3	1561	78	0.5599	0.6128	0.4637	0.3032	0.9205

CHR: chromosome; START bp: 5’end base pair; STOP bp: 3’end base pair; NSPS: number of SNPs genotyped for the gene; NPARAM: number of SNPs used for computing the gene wise statistics; p: SNP-wise mean p value. *p-value < 0.05; **p-value < 5 × 10^−4^.

### Agnostic GWAS of genetic variants associated with cardiac phenotypes and enrichment analysis of top genes on echocardiographic traits

**Figure 3:**
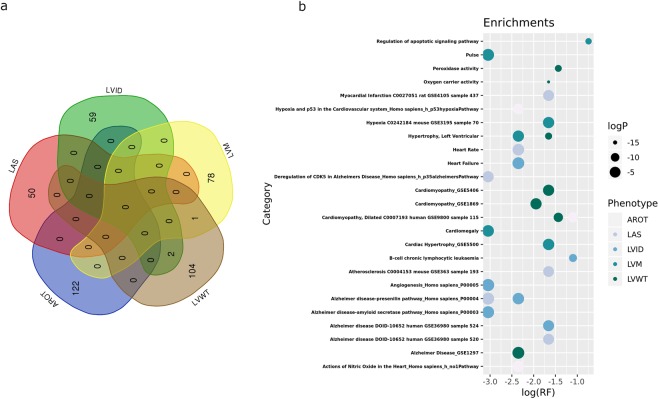
Results from gene-wise analysis of echocardiographic traits**. (a)** Venn diagram showing the overlap of top genes of the different echocardiographic traits analysed. **(b)** Plot summarising main results of the enrichment analysis of top genes from gene-wise statistics. RF (rich factor): number of genes from the input list included in each category divided by the total number of genes in the category.

Although we did not find any GWAS significant signal at SNP-level (p < 5 × 10^−8^) related with the analysed phenotypes, we observed several suggestive signals at the p < 10^−5^ level (Supplementary Tables 2–6), most of them intragenic (Fig. 2). For each genotype, we additionally performed a gene-level, ranking genes according to the MAGMA computed SNP-wise p-value, which uses the full spectrum of SNP-level meta-analysis results (Table 3 and Supplementary Tables 7–11). Overall, these top genes from MAGMA gene level analyses (p < 10^−3^) showed little overlap across phenotypes (Fig. 3a), with the exception of TECTB/ACSL5 locus for LVID and LVWT, genes associated to cholesterol and fatty acid metabolism respectively, and ZNF678, a zinc finger protein involved in immune response, for LVM and LVWT genes. Enrichment analysis (Fig. 3b, Supplementary Tables 12–16) suggested some processes underlying or related to one or more echocardiographic traits such as lymphocyte activity, cardiomyopathy, left ventricular hypertrophy or oxidative stress response Interestingly, enrichment analysis also suggested an over-representation of genes differentially expressed in AD samples versus control as well as genes involved in tau, presenilin and amyloid biology (APH1B, KLC3, KLC4, MAPK4, MAPK13;MAPK14, PPP2R5D, RBPJ, TCF7L1).

**Table 3:** Top ranked genes for each echocardiographic trait after genome wide association meta-analysis and gene-wise statistics calculation.

RANK	GENE	NSNPS	NPARAM	P
**AROT**				
1	*SMG6*	1594	114	1.35E-07
2	*METTL16*	1185	111	2.73E-07
3	*MNT*	1045	100	4.22E-07
4	*SGSM2*	1117	100	8.54E-07
5	*SYNE1*	3102	235	1.03E-06
6	*TSR1*	1015	98	1.32E-06
7	*SRR*	1010	90	1.61E-06
8	*SAXO1*	2109	154	1.68E-06
9	*ESR1*	2731	201	3.24E-06
10	*RRAGA*	1700	116	7.73E-06
**LAS**				
1	*AGMO*	2628	182	2.39E-05
2	*UBE2T*	774	84	7.69E-05
3	*NT5M*	1127	110	9.08E-05
4	*SLC35F2*	1431	96	1.11E-04
5	*SH2D6*	1194	115	1.46E-04
6	*E2F7*	1476	125	1.59E-04
7	*CAPG*	1281	118	2.03E-04
8	*PPP1R12B*	1264	97	2.40E-04
9	*ELMOD3*	1364	129	2.42E-04
10	*RAB39A*	1226	89	2.82E-04
**LVID**				
1	*SKA1*	1152	92	2.11E-07
2	*CXXC1*	1222	110	1.17E-06
3	*MBD1*	1277	106	1.36E-06
4	*CFAP53*	1308	111	1.50E-06
5	*CA12*	1222	112	4.40E-06
6	*USP3*	1296	85	1.73E-05
7	*FBXL22*	1078	65	2.12E-05
8	*ADRB2*	1296	134	5.56E-05
9	*ANKS6*	1496	137	6.32E-05
10	*LYRM9*	857	85	6.97E-05
**LVM**				
1	*MLF1*	1250	53	1.97E-07
2	*RSRC1*	2313	63	3.72E-07
3	*CLK1*	818	91	8.43E-06
4	*NIF3L1*	789	78	8.79E-06
5	*PPIL3*	807	82	8.82E-06
6	*GFM1*	1305	63	1.08E-05
7	*ORC2*	854	75	1.60E-05
8	*LXN*	1186	57	1.76E-05
9	*RNF20*	1444	102	1.93E-05
10	*BZW1*	868	104	2.21E-05
**LVWT**				
1	*ALDH7A1*	1467	154	1.22E-06
2	*OR52A5*	1732	132	3.69E-06
3	*MAPK13*	899	106	4.70E-06
4	*PHAX*	1392	141	7.73E-06
5	*OR52A1*	1735	141	8.10E-06
6	*OR52E2*	1688	115	8.21E-06
7	*GRAMD3*	1640	168	1.14E-05
8	*ZNF804A*	1589	89	1.28E-05
9	*OR52J3*	1697	112	1.68E-05
10	*PRSS38*	1094	88	1.85E-05

AROT: aortic root; LAS: Left Atrial Size; LVID: Left Ventricular Internal Dimension; LVM: Left Ventricular Mass; LVWT: Left Ventricular Wall Thickness; NSPS: number of SNPs genotyped for the gene; NPARAM: number of SNPs used for computing the gene wise statistics.

### Genetic correlation analysis between echocardiographic traits and AD

**Figure 4:**
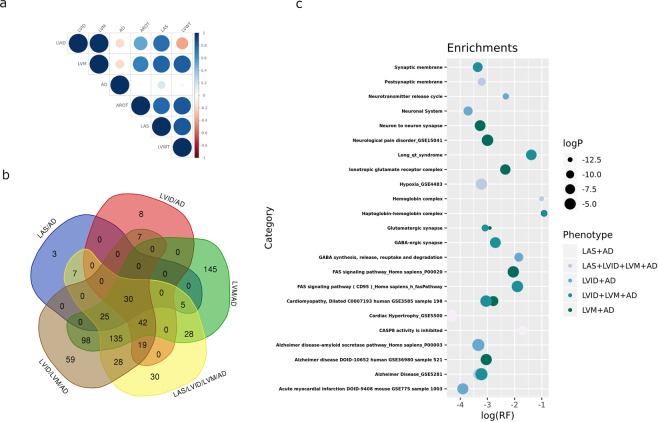
Results from the combined analysis of echocardiographic traits and AD. **(a)** Genetic correlations (rG) between echocardiographic traits and AD**. (b)** Venn diagram showing the overlap of top genes derived from gene-wise association analysis. **(c)** Enrichment analysis of top genes from meta-analysis of echocardiographic traits and Alzheimer’s disease. RF (rich factor): number of genes from the input list included in each category divided by the total number of genes in the category.

The association of gene variants related to echocardiographic measures with mental illnesses, the increasing interest in the vascular aspects of AD and availability of diverse AD datasets, prompted us to explore in more depth the relationship between the echocardiographic phenotypes and AD. We performed a genetic correlation analysis using the study dataset (comprised by 11,559 individuals with echocardiographic phenotypes) along with 12,730 AD cases and controls both from internal and publicly available databases. First, we estimated the proportion of variance, as a proxy of trait heritability, explained by all SNPs in each one of these traits^31^, which was higher for AD (0.38) than for the echocardiographic phenotypes (range: 0.17-0.36). Then, we looked for shared genetic loci across the genome between echocardiographic traits and AD using GREML analyses as suggested by the enrichment analyses. Specifically, we observed a positive correlation between AD and LAS (rG = 0.167, p = 0.0334), and negative correlations between AD and LVID (rG = −0.196, p = 0.0056), and AD and LVM (rG = −0.198, p = 0.0165); of note, LVM and LVID are the most correlated echocardiographic traits (rG = 0.988, p < 0.00001) (Fig. 4a). The sign of the rG estimates determines whether a direct or inverse relationship between the two phenotype traits is observed. Therefore, our results suggest that SNPs that increase the risk of AD tend to be associated with increasing LAS values. On the contrary, we found that SNPs associated with increased risk of AD tend to be associated with decreasing ventricular measures (or vice versa), in particular LVID and LVM.

Based on these findings we performed a SNP-wise meta-analysis by pooling Fisher association p-values of two or more phenotypes aimed at identifying genes contributing most to both heart measures and AD. Thus, we combined in these meta-analyses p-values for LAS&AD, LVID&AD, LVM&AD, LAS&LVID&LVM&AD and LVID&LVM&AD and calculated gene-wise statistics (Table 4, Supplementary Tables 17–21). We observed a large overlap of genes falling under the p < 10^−3^ threshold in the different meta-analyses performed, with a group of 30 genes consistently associated in all of them, including the CASP8/CASP10/CFLAR locus and the GABRR1 GABA receptor (Fig. 4b). Consequently, apoptosis related pathways driven by the *CASP8, CASP10* and *CFLAR* locus were persistently observed in the subsequent enrichment analyses (Fig. 4c, Supplementary Tables 22–25). We also observed an enrichment on genes present at the neuronal synapse such as glutamate (GRM5), cholinergic (CHRNA2) and GABA receptors (*GRIN2C, GABRR1, GABRR2* and *GABBR2*), teneurin (*TENM2*), calsyntenin (*CLSTN3*), or the cytoskeletal alpha-actin protein (ACTN2) for the diverse meta-analyses involving ventricular measures and AD.

**Table 4:** Top ranked genes for the combined analysis of echocardiographic traits and AD after genome wide association meta-analysis and gene-wise statistics calculation.

RANK	GENE	NSNPS	NPARAM	P
**LAS & AD**				
1	*NIF3L1*	467	46	1.03E-06
2	*PPIL3*	493	50	1.10E-06
3	*RSRC1*	1965	43	1.53E-06
4	*CLK1*	526	55	1.79E-06
5	*MLF1*	1068	33	2.16E-06
6	*ORC2*	486	40	4.67E-06
7	*BZW1*	596	65	1.04E-05
8	*SLC5A4*	1361	79	1.46E-05
9	*RFPL2*	1308	72	1.94E-05
10	*C22orf42*	1226	65	2.57E-05
**LVM & AD**				
1	*RSRC1*	1965	43	3.39E-11
2	*MLF1*	1068	33	3.82E-11
3	*NIF3L1*	467	46	5.98E-09
4	*GFM1*	1122	41	6.53E-09
5	*PPIL3*	493	50	6.66E-09
6	*ALDOB*	918	79	7.33E-09
7	*ZNF189*	886	80	8.96E-09
8	*MRPL50*	896	81	9.63E-09
9	*BAAT*	932	85	1.02E-08
10	*LXN*	1015	38	1.12E-08
**LAS & LVID & LVM & AD**				
1	*MLF1*	1068	33	3.25E-10
2	*RSRC1*	1965	43	4.65E-10
3	*ALDOB*	918	79	3.10E-09
4	*BAAT*	932	85	3.66E-09
5	*ZNF189*	886	80	3.72E-09
6	*MRPL50*	896	81	3.91E-09
7	*TMEM246*	1028	74	5.01E-09
8	*RNF20*	1056	61	6.62E-09
9	*NIF3L1*	467	46	1.82E-08
10	*PPIL3*	493	50	2.09E-08
**LVID & AD**				
1	*SKA1*	921	74	4.89E-06
2	*MLF1*	1068	33	6.39E-06
3	*ALDOB*	918	79	8.34E-06
4	*BAAT*	932	85	9.55E-06
5	*RNF20*	1056	61	9.63E-06
6	*ZNF189*	886	80	1.02E-05
7	*TMEM246*	1028	74	1.03E-05
8	*MRPL50*	896	80	1.06E-05
9	*RSRC1*	1965	43	1.17E-05
10	*CXXC1*	948	77	3.38E-05
**LAS & LVID & LVM & AD**				
1	*RSRC1*	1965	43	7.85E-10
2	*MLF1*	1068	33	8.85E-10
3	*NIF3L1*	467	46	1.95E-09
4	*PPIL3*	493	50	2.03E-09
5	*CLK1*	526	55	3.56E-09
6	*ORC2*	486	40	1.75E-08
7	*BZW1*	596	65	2.71E-08
8	*ALDOB*	918	79	3.51E-08
9	*ZNF189*	886	80	4.62E-08
10	*TMEM246*	1028	74	4.68E-08

AROT: aortic root; LAS: Left Atrial Size; LVID: Left Ventricular Internal Dimension; LVM: Left Ventricular Mass; LVWT: Left Ventricular Wall Thickness; NSPS: number of SNPs genotyped for the gene; NPARAM: number of SNPs used for computing the gene wise statistics.

## Discussion

Our study, based on data from 11,559 individuals free of cardiovascular disease, shows that variants affecting diverse genes involved in apoptosis regulation associate with echocardiographic phenotypes in humans. We have obtained these results by both hypothesis-driven and agnostic approaches. In addition, novel findings from the analysis of our GWAS also include previously unnoticed associations of variants in genes involved in cell proliferation, DNA replication and mRNA splicing with left ventricular morphology. Our data also suggest the existence of a set of genes, mainly related to apoptosis/inflammation signalling, whose variants are associated with both cardiac phenotype and AD.

Our hypothesis-driven analysis showed that caspases 8 and 10 and the regulatory *CFLAR* (cFLIP) gene are strong predictors of LVM. Furthermore, our hypothesis-free approach found that caspase dependent pathways were overrepresented among top genes associated with the LVM phenotype. These results support our a priori hypothesis that the apoptotic signalling influences heart morphology with potential impact on heart performance. Our hypothesis was based in previous experimental work, including our own, showing that deficiency^9,11^ or overexpression^32^ of key apoptotic genes altered normal cardiomyocyte differentiation and heart development independently of cell death. Indeed, although these genes are best known for their role in regulating apoptotic cell death, experimental evidences show that the same genes also regulate myocyte proliferation, inflammation and hypertrophy in the heart^9,11,33–35^. Because cell death is not a major event during heart development, and based on the above experimental work, we suggest that the relationship between the apoptotic genes and the cardiac phenotype might involve non-apoptotic functions.

In order to estimate the robustness of our GWAS analysis, we asked whether we had reproduced some signals already observed in previous genetic studies. Indeed, we found the already published link between *SMG6, TSR1* and *SRR* genes and AROT^16,17^, but failed to detect statistically significant associations for other gene variants, possibly due to the limited power of this study. Given the strong influence of age in echocardiographic parameters, the diverse population structure of the study cohorts could also affect our power in the meta-analysis, with signals not replicating in populations largely differing in their median age. However, our analysis demonstrated genetic association between variants of genes previously shown to influence heart function and cardiac hypertrophy in experimental models, such as *MLF1*^36^, which associates with LVM phenotype in our study, and *KCNIP2* (*KChIP2*)^37–39^ and *TRAF3IP2*^40,41^, which have also been associated with LVWT and LVM phenotypes respectively in humans and in this study as well. Also, from the top list of genes whose variants are associated to LIVD, *ANKS6* has been associated with heart malformations^42^.

The GWAS analysis also showed association of variants of a group of odorant receptors genes located in the 11p15.4 chromosomic region coding (subfamilies 51, 52 and 56) or in chromosome 16 (OR2C1) with LVWT and LVID. Expression of these genes in non-neural tissues is related to the control of different processes, including glucose and oxygen homeostasis or cell cycle control^43^, and has been shown to be involved in the regulation of cardiac function in rodent experimental models through interaction with fatty acids^44^. Therefore, our genetic results open the possibility that odorant receptors’ activity can influence cardiac function in humans. Interestingly, low OR expression has been found in the cortex of neuro-psychiatric patients^45^ and a genetic microduplication in the 11p15.4 region has been associated with familial intellectual disability and autism^46^.Unexpectedly, we found enrichment on AD related pathways for LAS, LVID and LVM that led us to explore comprehensively a possible link between AD and all these echocardiographic traits. Intriguingly, our results revealed for the first time a potential genetic link between AD and LAS, paired with a negative correlation between AD and either LVM or LVID. Interestingly, in line with our observation, recent reports found that LAS and LVID were independently associated to cognitive function in older adults, being predictors of cognitive decline after 14 years of follow-up^47,48^. The opposite direction of the correlation coefficients for atrial and ventricular measures could be related to the different development patterns of these chambers during embryogenesis^49^ and with the described strong association of left atrial size with long-term exposure to vascular risk factors, particularly high blood pressure and obesity. In fact, the CARDIA Brain MRI Substudy found association of higher left atrial volume in early adulthood with impairment of white matter integrity in midlife, but not for ventricular measures^50^.

A recent GWAS by our group on AD clinical endophenotypes revealed that vascular processes were the main causal mechanisms in pure AD^51^. But our findings are compatible to a broader definition of- dementia which includes other diseases such vascular dementia, fronto-temporal dementia or Lewy Body diseases among others. In fact, the rule is the presence of multiple pathologies in the brain of people with dementia including pervasive vascular lesions^52^. The link between dementia and cardiac conditions is not well understood. The old concept of cardiogenic dementia was based on the high incidence of cardiac dysrhythmias observed in patients with dementia due to vascular causes^53^. However, the relation between coronary heart disease (CHD) or HF and AD in epidemiological studies remains controversial, with some studies showing an association with cognitive impairment and dementia^54–56^ whereas others found no association^57,58^. The fact that both conditions are competing risks complicates the study of their relationship. Lower cardiac index levels are related to lower cerebral blood flow in older adults free of CVD^59^, but individuals with cardiac conditions that did not result in premature death might include many individuals chronically exposed to brain hypoperfusion due to reduced cardiac output that adaptively decreased cerebrovascular resistance through arteriolar dilatation. This kind of antagonistic pleiotropy between these phenotypes has been previously suggested by Beeri *et al*. after observing that better cognitive performance was associated with worse cardiac functioning in very elderly subjects^60^.

Moreover, whereas enlarged ventricular volume (LV hypertrophy) is a marker of diastolic dysfunction, LVM is also a marker of cardiovascular health, positively correlated with physical activity and cardiorespiratory fitness^61,62^. Population based studies have shown an inverted U-shaped association for LVM values and age, since they rise in adolescence and decline with increased age^63,64^. Furthermore, a U-shaped association between left ventricular ejection fraction (LVEF), a marker of systolic dysfunction, and abnormal cognitive decline has been reported, with increased dementia risk at the lowest and highest LVEF quintiles^65^.

To our knowledge, this is the first report analysing shared genetic factors between echocardiographic measures and AD. This method for estimating genome-wide pleiotropy has the advantage of being free of potential confounders determined by shared epidemiological risk factors such as high blood pressure or atherosclerosis. Our results show a negative genetic correlation for the ventricular measures LVID and LVM and AD, pointing to antagonist pleiotropic effects of shared genes by AD and LV cardiac measures, the main known functions of which are related to apoptosis (CASP8, CASP10, CFLAR, PHIP) and neurotransmission (GABRR1). Interestingly, some CASP8 variants have been previously associated with AD^66^, and the protein has been shown to be involved in amyloid related pathways and activated in both blood and brain cells from AD patients^1,67–69^. In our study, the GABA receptor GABRR1 was consistently associated in all meta-analyses involving heart traits and AD. Neurotransmission affects both cardiac and neuronal performance, and a few studies have examined synapse and neuron loss in AD brains and suggested that synaptic changes precede neuron loss^70,71^. In fact, GABA signalling is reduced in AD and other mental disorders such as dementia with Lewy bodies or frontotemporal lobar degeneration^72–74^. By contrast, augmented inhibitory GABAergic neurotransmission has been reported in animals models of HF and LVH through the involvement of the paraventricular nucleus of the hypothalamus^75,76^.

In summary, our GWAS data suggest the influence of gene variants affecting the apoptotic/inflammation signalling pathway on left ventricular morphology and cardiac function, uncover novel candidate gene variants regulating echocardiographic phenotypes and establish a genetic link between cardiac morphology alterations, mental illness and AD involving key genes in the regulation of apoptotic signalling that deserve functional assessment due to their diagnostic and therapeutic potential. These results should be replicated in larger datasets in order to confirm the observed pleiotropic effects for genes associated with echocardiographic traits.

## Supplementary information

Supplementary information

Supplementary data

## Data Availability

Full meta-analysis results for echocardiographic traits are available at Mendeley data (https://data.mendeley.com/, 10.17632/22jdjghnsp.1).
