# Supplementary material for: Genome Wide Meta-Analysis identifies common genetic signatures shared by heart function and Alzheimer’s disease
Source: Sci Rep. 2019 Nov 13;9:16665. doi: 10.1038/s41598-019-52724-2 (PMC6853976; doi:10.1038/s41598-019-52724-2)
Supplement: Supplementary file 1 — Supplementary information [file 41598_2019_52724_MOESM1_ESM.pdf]

Full title:

Genome Wide Meta-Analysis identifies common genetic signatures shared by heart function and Alzheimer's disease.

Short title:

Cardiac phenotypes and Alzheimer's disease

Authors

M.E. Sáez[1], A. González-Pérez[1], B. Hernández-Olasagarre[2], A. Beà[3], S. Moreno-Grau[2,4], I. de Rojas[2,4], G. Monté-Rubio[2], A. Orellana[2,4], S. Valero[2,4], J. X. Comella[4,5], D. Sanchís[3#], A. Ruiz[2,4#]

Coronary Artery Risk Development in Young Adults (CARDIA) Study – Cohort CARDIA is a longitudinal study cohort examining the aetiology and natural history of cardiovascular disease beginning in young adulthood (Hughes et al., 1987). The study was initiated in 1985 recruiting 5115 healthy black and white men and women aged 18-30 years. Follow-up examinations were performed at years 2, 5, 7, 10, 15, 20 and 25.

Cardiovascular Health Study (CHS) Cohort

The Cardiovascular Health Study (CHS) is a multicentre study of risk factors for development and progression of CHD and stroke in people aged 65 years and older (Fried et al., 1991).

Framingham cohort The Framingham Heart Study is a longitudinal community-based study initiated in 1948 in Framingham, Massachusetts, with the objective of identifying the common factors that contribute to CVD by following its development over a long period of time in a large group of participants who had not yet developed overt symptoms of CVD or suffered a heart attack or stroke(Dawber, Meadors, & Moore, 1951) . In 1971, the study enrolled a second-generation cohort of the original participants' adult children and their spouses(Feinleib, Kannel, Garrison, McNamara, & Castelli, 1975). We used available echocardiographic data of the offspring cohort at any of the examinations.

Multi-Ethnic Study of Atherosclerosis (MESA) Cohort

The Multi-Ethnic Study of Atherosclerosis (MESA) is a study of the characteristics of subclinical cardiovascular disease (disease detected non-invasively before it has produced clinical signs and symptoms) and the risk factors that predict progression to clinically overt cardiovascular disease or progression of the subclinical disease(Bild et al., 2002). MESA researchers study a diverse, population-based sample of 6,814 asymptomatic men and women aged 45-84 recruited from six field centres across the United States. Thirty-eight percent of the recruited participants are white, 28 percent African-American, 22 percent Hispanic, and 12 percent Asian, predominantly of Chinese descent.

The Alzheimer's Disease Neuroimaging Initiative (ADNI)

Data used in the preparation of this article were obtained from the Alzheimer's Disease Neuroimaging Initiative (ADNI) database (adni.loni.usc.edu). The ADNI was launched in 2003 as a public-private partnership, led by Principal Investigator Michael W. Weiner, MD. The primary goal of ADNI has been to test whether serial magnetic resonance imaging (MRI), positron emission tomography (PET), other biological markers, and clinical and neuropsychological assessment can be combined to measure the progression of mild cognitive impairment (MCI) and early Alzheimer's disease (AD). The ADNI study has three phases: ADNI1, ADNI GO and ADNI2. For up-to-date information, see www.adni-info.org.

The AddNeuroMed Study

AddNeuroMed was a public-private partnership for biomarker discovery and replication in Alzheimer's disease (Lourdusamy et al., 2012; Proitsi et al., 2014). It was designed as a multi-centre study in Europe with the first patient enrolled in January 2006 and the last in February 2008. The study protocol was planned for a baseline assessment visit with follow ups every 3 months for the first year, proceeded by annual visits that

continued through 2013. The study enrolled a total of 258 AD, 257 MCI and 266 controls, not all with complete data at each assessment.

The Alzheimer's Disease Genetics Consortium (ADGC)

The National Institute on Aging (NIA) Alzheimer's Disease Centres (ADCs) cohort includes subjects ascertained and evaluated by the clinical and neuropathology cores of the 29 NIA-funded ADCs (Naj et al., 2011). Data collection was coordinated by the National Alzheimer's Coordinating Center (NACC). The ADC cohort consists of autopsy-confirmed and clinically-confirmed AD cases, and cognitively normal elders (CNEs) with complete neuropathology data who were older than 60 years at age of death, and living CNEs evaluated using the Uniform dataset (UDS) protocol who were documented to not have mild cognitive impairment (MCI) and were between 60 and 100 years of age at assessment.

Multi-Site Collaborative Study for Genotype-Phenotype Associations in Alzheimer's disease and Longitudinal follow-up of Genotype-Phenotype Associations in Alzheimer's disease and Neuroimaging component of Genotype-Phenotype Associations in Alzheimer's disease (GenADA)

GenADA was a multi-site collaborative study, involving GlaxoSmithKline Inc and nine medical centres in Canada, including 1000 AD patients and 1000 ethnically-matched controls in order to associate DNA sequence (allelic) variations in candidate genes with AD phenotypes (Filippini et al., 2009; Li et al., 2008). The study consists of both retrospective and prospective data. Where possible, biological relatives with Alzheimer's (up to third degree relationship) and unaffected siblings of AD cases were also recruited.

The Mayo Clinic LOAD genome-wide association study

Subjects from the Mayo LOAD GWAS were selected from two clinical AD Case-Control series: Mayo Clinic Jacksonville (MCJ), Mayo Clinic Rochester (MCR)and a neuropathological series of autopsy-confirmed subjects from the Mayo Clinic Brain Bank (Carrasquillo et al., 2009). All subjects from the clinical series (MCJ and MCR) were diagnosed by a Mayo Clinic neurologist; all control subjects had a Clinical Dementia Rating score of zero at the most recent time of testing; all LOAD patients had a diagnosis of probable or possible AD according to the NINCDS-ADRDA criteria (McKhann et al., 1984). All ADs had definite diagnosis according to the NINCDS-ADRDA criteria and had Braak scores of ≥4.0. All non–AD Controls had Braak scores of ≤2.5; many had brain pathology unrelated to AD.

The Neocodex-Murcia study

The study includes 327 sporadic AD patients and 801 controls with unknown cognitive status from the Spanish general population collected by Neocodex (Antúnez et al., 2011; Gayán et al., 2010). AD patients were diagnosed as possible or probable AD in accordance with the criteria of the National Institute of Neurological and Communicative Disorders and Stroke and the Alzheimer's Disease and Related Disorders Association (NINCDS-ADRDA)(McKhann et al., 1984).

The Religious Orders Study and Memory and Aging Project (ROS/MAP) Study

The Religious Orders Study (ROS) is a longitudinal clinical-pathologic cohort study of aging and Alzheimer's disease (AD) from the Rush University that enrolled individuals

from religious communities for longitudinal clinical analysis and brain donation (A. Bennett, A. Schneider, Arvanitakis, & S. Wilson, 2012). Participants were enrolled from more than 40 groups of religious orders (nuns, priests, brothers) across the United States. Medical conditions are documented starting in 1994 by clinical evaluation or self-report. Alzheimer's Disease status was determined by a computer algorithm based on cognitive test performance with a series of discrete clinical judgments made in series by a neuropsychologist and a clinician.

The Memory and Aging Project (MAP) is a longitudinal, epidemiologic clinical-pathologic cohort study of common chronic conditions of aging with an emphasis on decline in cognitive and motor function and risk of Alzheimer's disease that began in 1997 and is run from Rush University (A. Bennett et al., 2012). This study was designed to complement the ROS study by enrolling individuals with a wider range of life experiences and socioeconomic status into a study of similar structure and design as ROS. The study enrols older individuals without any signs of dementia, primarily recruiting from continuous care retirement communities throughout north-eastern Illinois, USA. Diagnoses of dementia and AD are performed in an identical manner to the ROS study.

The Translational Genomics Research Institute (TGEN) study

The TGEN GWAS study included 643 late onset AD cases and 404 controls from a neuropathological cohort, and 197 late onset AD cases and 114 controls from a clinical cohort (Reiman et al., 2007).

List of Supplementary tables

- Supplementary_Tables_1-26.xlsx. Including the following tables:

S1. Alzheimer's disease datasets characteristics.

S2. Top genes (p<10-4) from the GWAS for AROT phenotype.

S3. Top genes (p<10-4) from the GWAS for LAS phenotype.

S4. Top genes (p<10-4) from the GWAS for LVID phenotype.

S5. Top genes (p<10-4) from the GWAS for LVM phenotype.

S6. Top genes (p<10-4) from the GWAS for LVWT phenotype.

S7. Gene-wise statistics (MAGMA) for AROT phenotype.

S8. Gene-wise statistics (MAGMA) for LAS phenotype.

S9. Gene-wise statistics (MAGMA) for LVID phenotype.

S10. Gene-wise statistics (MAGMA) for LVM phenotype.

S11. Gene-wise statistics (MAGMA) for LVWT phenotype.

S12. Enrichment analysis of 200 top genes from AROT association analysis.

S13. Enrichment analysis of 200 top genes from LAS association analysis.

S14. Enrichment analysis of 200 top genes from LVID association analysis.

S15. Enrichment analysis of 200 top genes from LVM association analysis.

S16. Enrichment analysis of 200 top genes from LVWT association analysis.

S11. Gene-wise statistics (MAGMA) for LVWT phenotype.

S17. Gene-wise statistics (MAGMA) for the meta-analysis of AD and LAS phenotypes.

S18. Gene-wise statistics (MAGMA) for the meta-analysis of AD and LVID phenotypes.

S19. Gene-wise statistics (MAGMA) for the meta-analysis of AD and LVM phenotypes.

S20. Gene-wise statistics (MAGMA) for the meta-analysis of AD, LAS, LVID and LVM phenotypes.

S21. Gene-wise statistics (MAGMA) for the meta-analysis of AD, LVID and LVM phenotypes.

S22. Enrichment analysis of 200 top genes from AD and LAS meta-analysis.

S23. Enrichment analysis of 200 top genes from AD and LVID meta-analysis.

S24. Enrichment analysis of 200 top genes from AD and LVM meta-analysis.

S25. Enrichment analysis of 200 top genes from AD, LAS, LVID and LVM meta-analysis.

S26. Enrichment analysis of 200 top genes from AD, LVID and LVM meta-analysis.

- Supplementary_Tables_27-36.xlsx. Including the following tables:

S27. Enrichment analysis for AROT at different p values thresholds (Webgestalt).

S28. Enrichment analysis for LAS at different p values thresholds (Webgestalt).

S29. Enrichment analysis for LVID at different p values thresholds (Webgestalt).

S30. Enrichment analysis for LVM at different p values thresholds (Webgestalt).

S31. Enrichment analysis for LVWT at different p values thresholds (Webgestalt).

S32. Enrichment analysis for AROT at different p values thresholds (enrichR).

S33. Enrichment analysis for LAS at different p values thresholds (enrichR).

S34. Enrichment analysis for LVID at different p values thresholds (enrichR).

S35. Enrichment analysis for LVM at different p values thresholds (enrichR).

S36. Enrichment analysis for LVWT at different p val
